# How do urban block built environments affect older adults’ walking activities and health effects: a case study in Nanjing, China

**DOI:** 10.3389/fpubh.2024.1479305

**Published:** 2024-09-30

**Authors:** Congjian Chen, Yang Cao, Guangfu Xu, Qing Zhong, Bing Chen

**Affiliations:** ^1^College of Construction Engineering, Jiangsu Open University, Nanjing, China; ^2^Science and Technology Department, Jiangsu Open University, Nanjing, China; ^3^Nanjing Gardening-Landscaping Economic Development Limited Liability Company, Nanjing, China

**Keywords:** built environment, older adults, walking activities, health effect, multi-layer linear model

## Abstract

**Introduction:**

Research on the relationship between microscale built environments and physical activity among older adults in densely populated old urban areas with high aging rates is scarce. Particularly, the relationship between urban block land-use pattern and older adults’ walking activities have not yet been completely understood.

**Methods:**

This study examined the daily walking habits and socioeconomic attributes of older adults in 17 blocks with different built environment characteristics in Nanjing City, China. A multi-layer linear model was used to quantitatively analyse the mechanism underlying the effects of various factors on the health of older adults.

**Results:**

The result shows significant positive correlation between neighborhood walkability and the enhancement of walking frequency and activity. For every 10% increase in pedestrian road connectivity and land use, the walking activity volume increased by 22.4 and 12%, and the BMI increased by 9.1 and 7.3% toward the standard range, respectively. For every 10% increase in distance between residence and plaza and park, the walking activity volume decreased by 5.4 and 3.2%, and BMI decreased by 9.9 and 6.3%, respectively.

**Discussion:**

For every 10.4% increase in land mixed-use rate and per capita green area, BMI increased by 19.4%. Furthermore, higher household income and number of family members have significant correlation with walking activities and health effects. Moreover, the block-scale built environment and walking activities jointly affected health, with a cross-functional relationship between multiple factors.

## 1 Introduction

Currently, more than half of the world’s population lives in urban areas (1). Urbanization has significantly improved people’s living environments; however, urban lifestyles such as excessive reliance on motor transportation, increased commuting time, and prolonged office sitting have indirectly led to a lack of physical activity (1). Regular sports activities can be considerably beneficial for the physical and mental health of older adults (2). Many studies have shown that moderate physical activity can not only significantly improve older adults’ abilities and reduce the risk of heart disease, diabetes, and other diseases, but can also help reduce the risk of depression and anxiety (3). Short distance walking is considered a preferred outdoor physical activity among older adults (4). Many studies posit that walking activities are not only influenced by individual physiological and psychological characteristics but also by their environment (5, 6). Among these, the urban built environment has proven to be a key factor affecting pedestrian activities (7, 8). Therefore, street space is crucial for its commuting, leisure, social, and exercise purposes (9, 10). Evidence suggests that streets with suitable pedestrian environments can encourage and support older adults to engage in more physical exercise and social activities outside (11, 12). Therefore, assessing the walking environment of older adults on urban streets is important for building cities that are older adult friendly and to further stimulate future policy-making (13).

Previous studies have shown that moderate intensity walking activities are easier to integrate into the daily lives of older adults compared to other high-intensity physical activities (14). As physical function continues to decline, walking has become one of the most common and beneficial physical activities in the daily lives of older people. Older adults can partake in two types of walking activities. The first derives from necessary daily travel activities such as shopping, picking up and dropping off children, and social interaction. The second involves spontaneous travel activities such as walking dogs through physical exercise and social interaction. Numerous research studies have focused on older people’s walking behavior from a health perspective. The content encompasses multiple fields, such as public health, sports science, medicine, geography, and urban planning, with a focus on exploring the impact of the built environment and individual factors on walking activities (15, 16). Research generally suggests that as older people age, the spaces in which they engage in activity shrink, and neighborhood spaces become their main activity spaces (17).

Research on residents’ walking activities and health has focused on the built environment. Various factors have been used to measure built environments. Cervero and Kockelman categorized the built environment into three important dimensions (3Ds): density, diversity, and design (18). Handy et al. proposed six built environment characteristics: density, degree of mixed land use, street connectivity, block scale, esthetics, and regional structure (19). Scholars have explored the characteristics of built environment elements from four perspectives: functionality, safety, esthetics, and destination (20). From the perspective of research scale, the impact of the built environment on physical activity can be divided into three dimensions: macro, meso, and micro. The macro level focuses on the entire city, with research focusing on the impact of factors such as urban form expansion and infrastructure layout on the physical activity of the population (21). The meso level refers to the composition range of one or more urban blocks, focusing on the impact of density, land-use mixing degree, street connectivity, and other factors on the physical activity of the population (22). The micro level focuses on buildings and their site selection, including the impact of venue design, street scale, and public facility distance on the physical activity of the population (23).

The impact of public service facilities on the walking activities and health effects of older adults is a complex multidimensional issue that involves multiple disciplines, including urban planning, public health, and social economics (24). With the gradual maturity of health circle model research, quantitative statistical analysis has been conducted on the living environment of the older adult population. It is believed that the main factors affecting residents’ health include population agglomeration, socio-economic attributes, diseases, and the supply of medical and health service facilities (24). In terms of mental health, factors such as life satisfaction and happiness can also have an impact on it (25). Some scholars in China have conducted case studies on the relationship between public service facilities and the health of the older adult population, including identifying the scale, type, coverage, and accessibility of public service facilities, and recording the satisfaction and optimization suggestions of facility use. They unanimously agree that public service facilities have a significant impact on the behavior and health of the older adult population (26, 27). However, existing research has been limited to a specific type of facility and lacks an overall study on the public facility system that affects the health behavior of older adults. Case studies on the interaction between multidimensional factors such as public service facilities, roads, built environments, and buildings are especially lacking.

The impact of a slow traffic environment on individual physical activities of residents in the neighborhood not only covers the objective material function usage level but also includes subjective perception differences of the built environment caused by residents’ own socioeconomic level differences. In terms of measurement indicators, the objective pedestrian index and subjective environmental perception of the built environment at the block scale have received significant attention from relevant research (24). In existing research, the objective walking index is generally composed of indicators such as residential density, street connectivity, and land-use mix, which are stacked with different weights. The walking environment characteristics of highly connected streets, high residential density, and mixed land use are conducive for increasing the physical activity of adults (25). Research has shown that higher quality of neighborhood walking environments is associated with higher enthusiasm for walking and significant increases in leisure physical activities among older adults (26). Some studies have introduced a subjective walking environment perception evaluation scale based on an objective walking index to quantitatively measure residents’ perception levels of walking facility convenience, spatial barriers, attractiveness, and safety (27). Studies have found that a good perception of the walking environment promotes residents’ physical activity (28). Specifically, the better an adult’s perception of street neighborhood connectivity, the higher the probability of choosing walking or cycling as the mode of transportation (29). Moreover, stronger perceptions of walkability and esthetics of public open spaces are associated with higher level of walking activity and ease of reaching the recommended level of healthy physical activity among residents (30). However, the perception level of safety aspects, such as traffic and crime, may affect residents’ willingness to engage in outdoor activities, thereby affecting their transportation and leisure activities (31). The better the perceived distance of residents toward public transportation stations or destination, the more likely they are to increase opportunities for walking or cycling (32) and frequency of leisure physical activities (33, 34).

Health inequality is among the popular topics of concern in the sociology field, and walking activities have received attention as a mediating variable affecting residents’ physical health (35). Scholars are constantly exploring the interaction mechanism between socioeconomic status and residents’ walking activities, attempting to narrow the gap in walking activity levels among residents of different social classes (25, 27, 36). However, previous studies have found that with societal development and the overall improvement of people’s living standards, this level of inequality has not gradually decreased, and walking activities and health levels still maintain a sustained positive correlation with socioeconomic status (30, 32, 37).

Some planning scholars believe that the neighborhood environment includes three independent dimensions: street living environment, social neighborhood environment, and residential living environment (38). The block-scale built environment examined in this study refers mainly to a combination of street space elements that meet the daily living needs of residents, covering various life service facilities, road traffic environments, and public space elements, such as green spaces (39). Therefore, this study posed the following research questions: (1) What specific factors in a block-scale built environment have a significant impact on older adults? This study focuses on analyzing how the built environment affects walking willingness, which in turn affects walking behavior patterns and ultimately affects the health of older adults. (2) Do the daily habits and socioeconomic background of older adults impact their health? Do physical activity and environmental factors have cross-effects on the health of older adults? (3) Is the impact of block-scale built environments on walking among older adults universally applicable? Are the effects of different social backgrounds on older adults consistent? What type of older population is more susceptible to the impact of the built environment in a neighborhood?

Overall, existing research on the impact of urban built environments on older adults’ walking activities does not consider the actual impact on activity space and location from the perspective of residents’ daily activities, and it does not compare the differences in the impact of built environments within different activity space ranges (40). Considering this literature gap, this study focuses on the older adult population, with the planning goal of guiding healthy lifestyles and improving health levels and quality of life. From a health perspective and based on the connection between supply and demand, it examines the impact of built environment characteristics on daily walking activities. The research attempts to provide a basis for further adjustment and optimization of the structure, function, and quality of urban built environment elements. From the perspective of older adults’ adaptability to the environment, improving the aging performance of built environment elements can somewhat compensate for the loss of physical function in the older adult population, support their physical activity, and extend their independent living time. In addition, this study constructs a model framework of “built environment subjective perception physical behavior health effects,” revealing the matching relationship between walking activity and participation in activities among the older adult population under specific environmental conditions, as well as summarizing and verifying the health effects of built environment elements.

## 2 Data and methods

### 2.1 Study area

**Figure 1:**
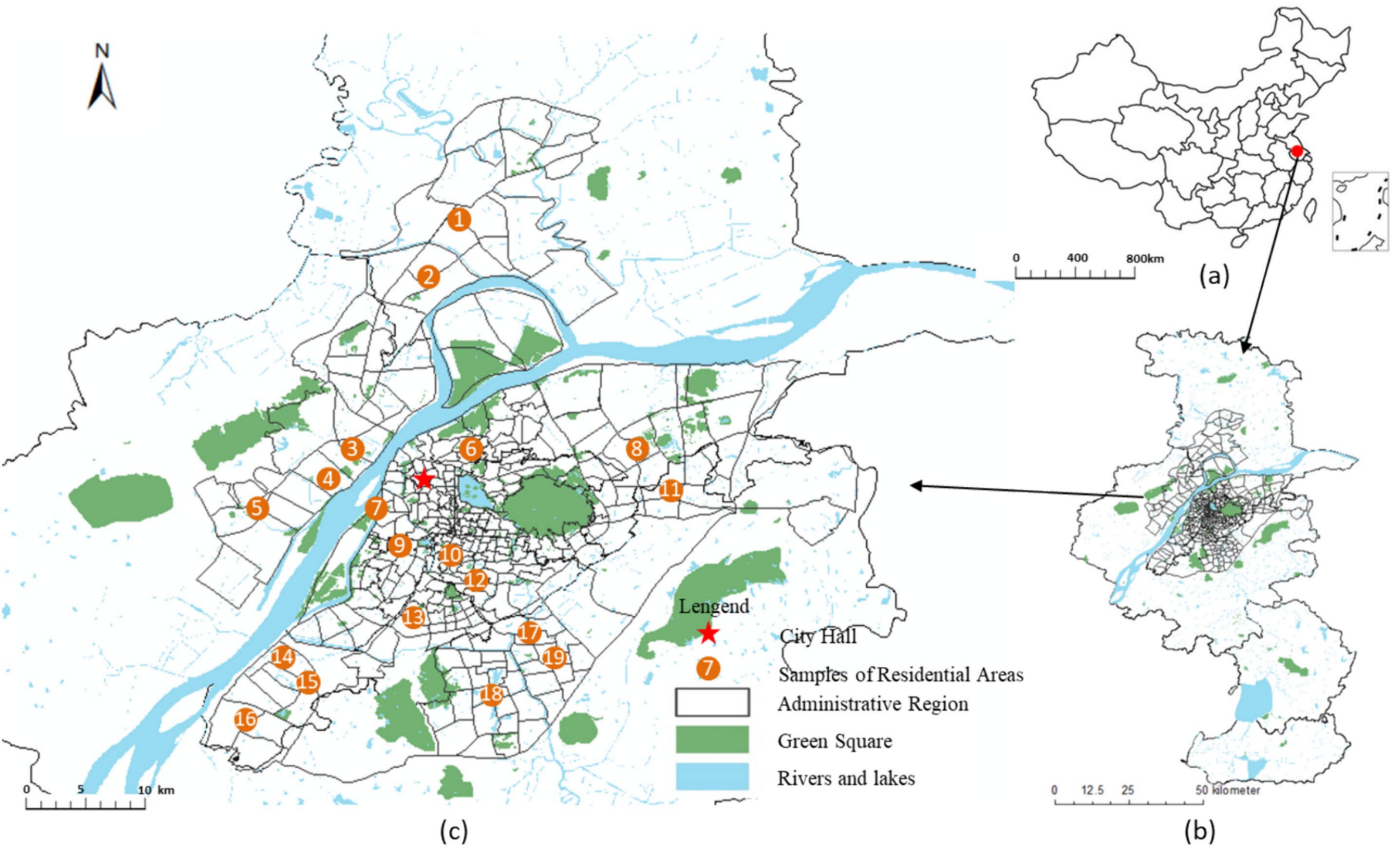
Location of Nanjing and the study area. **(A)** China; **(B)** Nanjing; **(C)** The study area.

This study examined the main urban area of Nanjing City, under the jurisdiction of Yangzhou City, in Jiangsu Province, with a population of approximately 330,000 and a total area of 52.12 km^2^ (Figure 1). With the rapid development of the local social economy in recent decades, significant changes have occurred in urban structures and socioeconomic environments, such as rapid population agglomeration in central urban areas. Many large-scale restructuring and renovations have been carried out in the built environment at the block scale. Geographical hierarchies in Chinese cities are generally divided into three administrative levels: urban, district, and block. Based on previous studies, this study regarded blocks as the basic units of research (29). A block is the smallest administrative unit as the basic unit of China’s urban census. The delimitation of urban blocks in China is mainly based on the population size of the unit, and the corresponding public service facilities and resource allocation are also based on this standard, which meets residents’ daily lives and travel needs (41, 42). However, the differences in social and economic attributes among residents in different blocks are much greater than those within blocks (43). Unlike European countries, cities in China are generally larger in scale, and the spatial heterogeneity of urban neighborhoods is more evident. Heterogeneity is mainly manifested in the supply of public service facilities, land-use structures, and the population socioeconomic attributes of streets. Therefore, an in-depth analysis of the differential characteristics of the impact of built environments on residents’ health in small-scale units within cities is required.

Nanjing was selected for the case study. Nanjing is the capital city of Jiangsu Province, with a total land area of 6587.1 square kilometers and an urban built-up area of 868.3 square kilometers. In 2019, Nanjing had jurisdiction over 11 municipal districts, 94 streets, and six towns. In 2019, the total population of Nanjing was 9.282 million, the urban population was 8.066 million and the urbanization rate reached 86.9%. The urban built-up area of Nanjing presents a circular spatial pattern. The inner old city covers an area of 172.3 square kilometers, including some streets in Gulou District, Qinhuai District, and Xuanwu District. The population distribution in the old urban area of Nanjing is dense, and the city is in the late stage of urbanization transformation and development. The main urban area around the old city covers an area of 372.8 square kilometers, including the Jianye District, Qixia District, Yuhuatai District and other streets. The outer new urban area covers an area of 323.2 square kilometers, including the Jiangbei New Area, Jiangning District, and other streets.

We specifically selected the main urban area of Nanjing as the research scope. Nanjing is one of the cities with the highest aging rate in China, and the main urban area has the highest degree of aging in Nanjing. Its proportion of older adults’ population has significantly exceeded the city’s average level. Simultaneously, the new construction and development in the main urban area of Nanjing is relatively small, the built environment quality is high, and the infrastructure configuration is complete (Table 1).

**Table 1:** Basic information of the surveyed communities.

Block type	Residential community	Built year	House price/yuan	Distance from the city center /km	Number of valid questionnaires
Old housing concentration area	Nanxin	1981	10,000–20,000	0–5	67
	Ladefangsi	1991	Below 20,000	0–5	62
	Tongfangyuan	1987		0–5	78
	Cuihuyiqu	1983	20,000–30,000	0–5	89
	Fantongyuan	1985	Below 20,000	5–10	82
	Decaiti	1993	10,000–20,000	5–10	63
Affordable housing concentration area	Yuehujia	2001		5–10	59
	Zhongnanhua	2005	20,000–30,000	5–10	82
	Wankejiazu	2007	Below 20,000	10–15	63
	Shimaojia	2004	10,000–20,000	10–15	59
	Yunxiyayuan	2008		10–15	86
Improved housing concentration area	Tongtiange	2012	Below 20,000	10–15	78
	Jiatianxia	2014		10–15	63
	Yuxiguoji	2016	20,000–30,000	5–10	72
	Lizhangfu	2016	Below 20,000	10–15	82
	Kongtianyuan	2019	20,000–30,000	5–10	72
	Shimaojia	2018	Below 20,000	10–15	82

**Figure 2:**
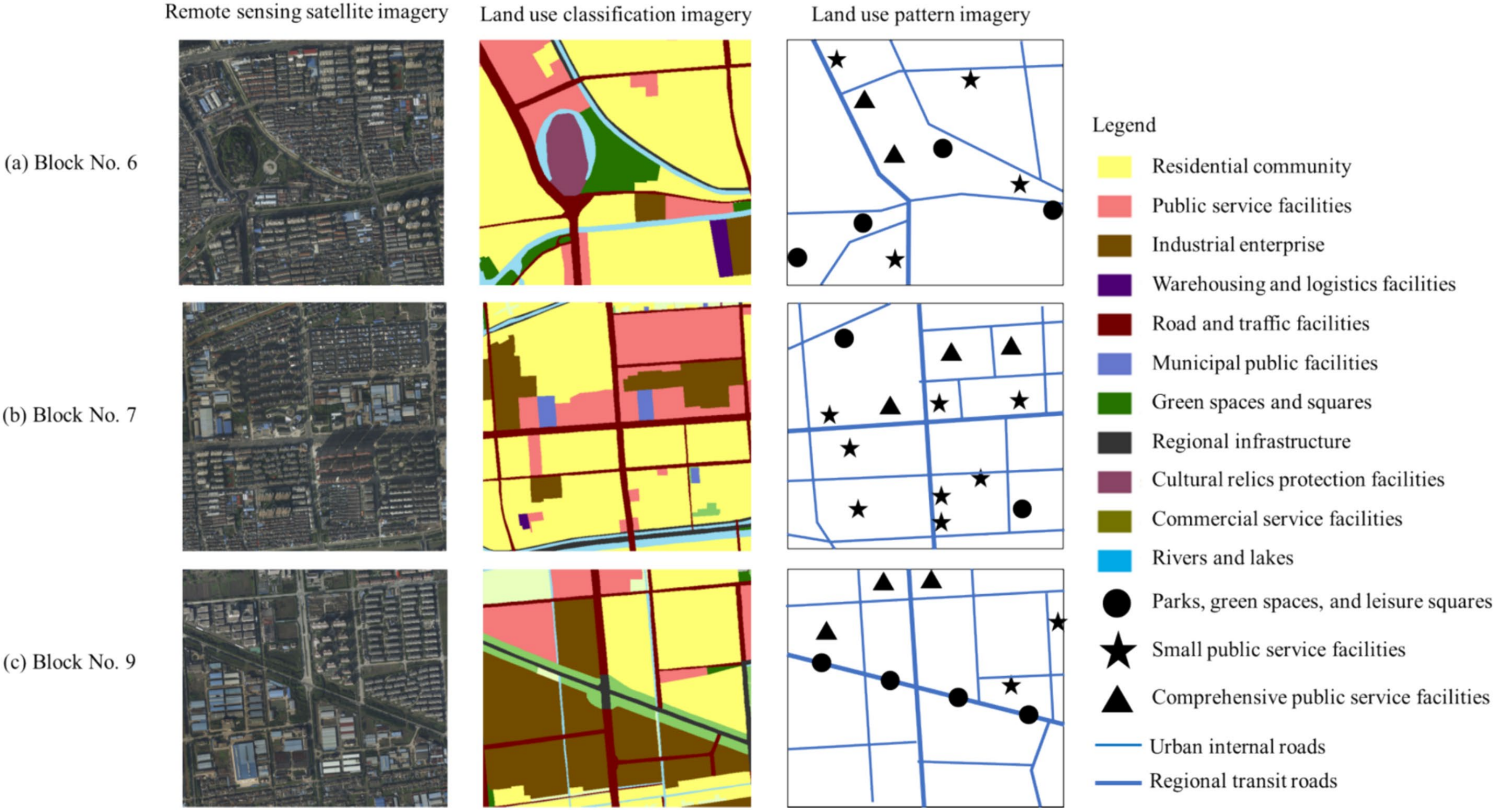
Typical block remote sensing images, land use types, and land use pattern maps. **(A)** Block No.6, **(B)** Block No.7 and **(C)** Block No.9 are the three blocks within the study area shown in Figure 1.

Figure 2 shows three typical blocks within the study area, The land cover data products for the year 2023 were obtained from the Data Center for Resources and Environment Sciences, Chinese Academy of Sciences (https://www.resdc.cn/ accessed on 12 May 2023.). These data products were derived from remote sensing satellite imagery and were manually interpreted by professional personnel and visually interpreted using machine learning techniques. The overall accuracy of the data exceeds 90%, and it includes 11 major land-use categories: residential community, public service facilities, industrial enterprise, warehousing and logistics facilities, road and traffic facilities, municipal public facilities, green spaces and squares, regional infrastructure, cultural relics protection facilities, commercial service facilities and rivers. For validation, 200 sample points containing detailed land classes from medium- to high-resolution remote sensing images were selected using Google Earth. The results showed that the overall accuracy of the data exceeded 94.3%, with a Kappa coefficient above 0.96, meeting the accuracy requirements of the study. We have drawn a classification map of the current land use status in the research area, and classified the land use into various types such as residential land, industrial enterprise land, green space and square land, road and transportation facility land, and public service facility land. Then, we extracted the core and important land features based on the actual land use situation of 17 surveyed blocks, and constructed a block land-use pattern map. The land-use pattern map mainly contain three parts: the organization pattern of road networks at different levels in the block, the layout pattern of public service facilities at different levels, and the spatial layout pattern of parks, green spaces, leisure squares. These contents correspond to quantitative indicators such as land use mix, facility diversity, road connectivity, and facility accessibility.

### 2.2 Variables and indicators

This study focused on the factors of built environment features, individual socioeconomic context, and daily behavioral habits. The data structure contained the variables at both the block and individual levels. Based on existing research results (32, 36, 42, 43), this study constructs a set of factors that affect the health effects of the built environment, including two types of built environment variables: spatial characteristics and environmental factors. Among them, spatial characteristic variables mainly include land distribution density, land use mix, road network layout, and accessibility of public service facilities. Environmental element variables mainly include walkability, green and open spaces, and site facility conditions. The relevant data mainly originates from the Nanjing Geographic Information GIS database, as well as the field investigation conducted by the research team on the built environment of typical communities.

#### 2.2.1 Dependent variable

The explanatory variables in this study were walking index (Y1) and health level (Y2) of older adults. The walking index evaluation included two levels: the walk score for each point and the walk score for the city neighborhood. This study used a single-point walking index for evaluation. This method is aimed at evaluating a specific point in space, focusing on the distribution of various facilities within a suitable walking range and considering the impact of the walking environment, such as travel distance, intersection density, and block length. The basic score was calculated based on the accessibility of the facility and was corrected using distance and environmental data to obtain a single-point walking index score (44, 45). The interpreted variable in this study was body weight. Data were measured by body mass index (BMI), an internationally accepted obesity degree index. The BMI value was obtained by dividing the square of the height in meters by the weight in kilograms. This is currently a common international standard for measuring body weight and fitness (46). According to the BMI indicators provided by the World Health Organization (WHO), which are suitable for Asian ethnic characteristics, we set the scoring standard using a five-point Likert scale. The numerical interval of 18.5–23.9 is defined as the standard body shape, and five points are assigned. The intervals of 15.3–18.5 and 24–27 were defined as slightly unbalanced shapes, and four points were assigned. The intervals of 13.7–15.3 and 28–30 were defined as obviously unbalanced shapes, and three points were assigned. The intervals of 10.4–13.7 and 30–32 were defined as highly unbalanced shapes, and two points were assigned. An interval of less than 10.4 and more than 32 was defined as an extremely unbalanced shape, and one point was assigned. Overall, the higher the score, the better the health of the residents.

#### 2.2.2 Independent variable 1: block-scale built environment

**Figure 3:**
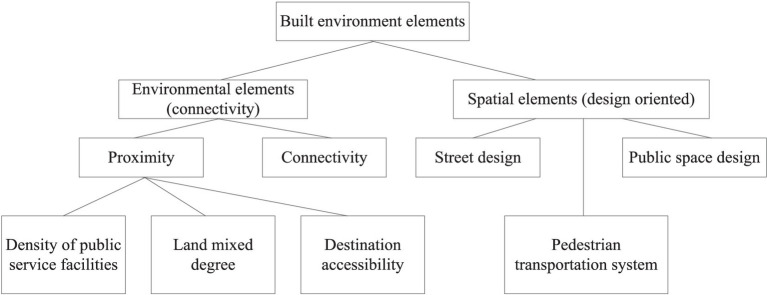
Classification of block-scale built environment elements.

Research has shown that good public service facility allocation, open street connectivity, and mixed land use can effectively enhance residents’ daily physical activity willingness and activity frequency (30). Existing research has primarily measured street walkability using pedestrian street length, area, intersection density, and other indicators. This shows that good street connectivity can shorten commuting distances and increase walking probability (31). This study selected 17 residential blocks in a central urban area and measured the organizational characteristics of the built environment features from five aspects: land use, pedestrian streets, public service facilities, green spaces, and public transportation (Figure 3). Specifically, the land-use level was measured by the plot volume ratio and land-use mix index in the residential area (31). Pedestrian street mileage, density, and connectivity were used to measure block openness. The number and accessibility of public service facilities within 1 km^2^ was used to measure the level of public services. The number and accessibility of green parks within 1 km^2^ were used to measure the green space service levels. The number and accessibility of public transit facilities within a block were used to measure public transportation convenience (47).

#### 2.2.3 Independent variable 2: socioeconomic attributes

Socioeconomic attributes of residents have a significant impact on their health. Studies have shown that people with higher levels of education in their families generally have higher health awareness (43). Older adults with a complete family structure can receive companionship from spouses and children, thereby stabilizing their health. Higher household income makes it easier to obtain healthy food and a good living environment and maintain good health (43). Therefore, the indicator system selected three individual characteristic indicators: education level, marital status, and family income. These characteristics have an indirect impact on residents’ health levels, as control variables.

#### 2.2.4 Independent variable 3: daily living habits

Daily routines and dietary habits are important factors affecting residents’ health (BMI). Research has shown that unhealthy and irregular lifestyle habits can lead to disorders in the human metabolic system and hormone secretion, thereby affecting health (44). This study obtained data on individual daily dietary habits, physical exercise habits, and other factors from residents’ questionnaire surveys. Dietary habits were divided into four categories: balanced nutrition, vegetarian diet, meat-based diet, and salt preference, oil, and sugar. Healthy dietary indicators provided by the International Dietary Organization were rated on a four-point scale, with higher scores indicating healthier dietary habits (40). We used frequency as an indicator to measure walking fitness habits, which were classified into four categories: never, occasionally, frequently, and daily.

### 2.3 Data source and processing

The data included environmental factors of block construction, socioeconomic attributes, and daily living habits. Built environment data were obtained from internet electronic maps and field research, while socioeconomic data and residents’ daily living habit data were derived from residents’ health questionnaire surveys. A multi-layer linear model can handle samples with uneven distributions. To analyse and verify the impact of high-level variables, we removed sample data with incorrect or missing attributes. The final number of samples included in the model was 3,239, involving 17 neighborhoods. The basic statistical information for the samples is presented in Table 2. We conducted spatial mapping and integration of the spatial and attribute information obtained through research using a GIS platform to achieve a spatial linkage between the walking behavior of older adults and built environment. Moreover, we desensitized and deleted the personal information of the investigated groups to ensure that the private data of individual residents (including personal name, ID card number, and home address) were not included. Furthermore, the data of the institute was comprehensive and complete. Specifically, we replaced the name with a number in the basic attribute information of residents, partially encrypting the ID number, and simplifying the family address information. Residents’ walking activity data was used only for this study and not for commercial use. Furthermore, the research conclusion did not involve key sensitive information, such as residents’ personal names and home addresses.

**Table 2:** Basic information of independent variables.

Variable content	Variable index	Measure method/Descriptive statistics
**Built environment features**		
Land-use	Residential density	The ratio of overall floorage in land-use area
	Land-use mix	Information entropy (EI) of land-use types in search range to reflect land-use mixing degree
Pedestrian street	Street mileage	Total mileage of pedestrian streets within: less than 1 km (43.8%); 1–3 km (26.4%); 3–5 km (24.1%); more than 5 km (5.7%)
	Street density	The ratio of the pedestrian streets number to the streets’ total number
	Street connectivity	The ratio of the pedestrian street intersections to the total number of streets
Public service	Number of public service facilities	Number of public service facilities within 1 km: none (17.5%); 1–3 (54.2%); 3–5 (17.8%); over 5 (10.5%)
	Accessibility of public service facilities	Average distance between residence and nearest public service institution
Green space	Number of green parks	Presence (42.4%) or absence (57.6%) of green space within 1 km
	Accessibility of green parks	Average distance between residential area and nearest green space in the neighborhoods
Public transportation	Number of public transit facilities	Number of public transport facilities within 1 km
	Accessibility of public transit facilities	Average distance between residential area and nearest public transport station
Individual conditions	Age	Less than 24 (0.4%); 25–44 (2.9%); 45–59 (19.2%); over 60 (77.5%)
	Gender	Male (62.5%); Female (37.5%);
	Education	Primary school and below (72.9%); junior middle school (15.6%); senior middle school (9.9%); bachelor’s degree or above (1.6%)
	Marital status	Married (47.3%); widowed (21.9%); unmarried (30.8%)
	Household income	Family annual income: less than 5,000 yuan (46.6%); 5,000–10,000 (27.5%); 10,000–50,000 (18.1%); above 50,000 (7.8%)
Daily life habits	Dietary habits	Nutritional balance (23.3%); mainly meat (12.6%); mainly vegetables (39.7%); excess salt, oil, sugar (24.4%)
	Exercise habits	Never (22.7%); occasionally (34.2%); frequently (17.9%); every day (25.2%)

Specifically, in the questionnaire design stage, the questionnaire conducted several rounds of discussions within the research team and sought the opinions of medical psychology and medical ethics experts. Ethics approval was obtained from the Medical Ethics Committee of Nanjing Health Bureau. At the beginning of the symposium, the medical ethics experts were provided with a written research content schedule and a verbal explanation of the research purpose. Then we received the ethical committee’s approval. This study drew on the research specification of the existing achievements (42, 43) and the study was conducted in accordance with the 1995 Helsinki Declaration (42). In the questionnaire survey stage, we distributed 100 questionnaires for pre-study and all subjects gave their informed consent for inclusion before they participated in the study to ensure that the content of the questionnaire could be accepted by respondents. The specific instructions were also included in the text of the questionnaire that ‘we confirm that the results will be only used for academic statistical research rather than other use in any form. Your participation is anonymous. If you have any discomfort during the questionnaire process, you can interrupt it at any time. We promise not to report or disclose your personal information in any way’. Participants gave consent to participate in the study on the survey of individual socioeconomic background, public service facilities evaluation, and built environment assessment. Notably, to protect the privacy of respondents, their name attributes were actively removed during the data processing. This paper mainly studies the common characteristics of respondents’ daily walking activities with similar socioeconomic background and built environment conditions.

We have fully considered the social diversity requirements of demographics in our data collection work. Based on social stratification theory, social stratification refers to the phenomenon of stratification or differentiation among social members and groups due to different ownership of social resources (31, 32). Especially for older adults, who are generally in retirement status and do not need to work, their residential address is basically fixed (33, 34). We conducted a questionnaire survey in 17 districts of Nanjing city, taking into account the heterogeneity and diversity of different types of older adults when selecting the sample size. In major cities in China, the built environment is divided into different functional zones based on different functional positioning and development models. There is heterogeneity in land use structure, built environmental elements, and transportation network systems among different functional zones. The 17 blocks selected for this study have differentiated built environment attribute characteristics, including geographical location, housing type, construction time, and housing prices. These indicators have been explained in Table 1, which could reflect the socio-economic diversity of the survey sample population. In China, the definition of older adults usually refer to citizens over 60 years old. This standard was determined based on the actual social situation in China and the trend of population aging (24). In addition, the WHO defines older adults as those aged 60 and above, with ages 45–59 referred to as middle-aged or early old age, and ages 60 and above referred to as old age (25). Among the surveyed population in this study, 77.5% were over 60 years old. We conducted a questionnaire survey on this group to obtain targeted behavioral and attribute information of the older adult population.

Before conducting model validation, we need to conduct reliability and validity analysis on the questionnaire. Reliability is used to analyse the reliability, stability, and consistency of a questionnaire (35). Validity indicators are used to test whether the descriptions of each item meet the expected research objectives of this article. We usually use Cronbach’s Alpha coefficient and KOM value to represent the reliability and validity of the questionnaire, respectively. If the alpha value is above 0.8, it indicates high reliability; if the KOM value is above 0.7, it indicates good questionnaire validity. This study analyses the reliability and validity of the questionnaire through a model (see the results in Table 3). The Cronbach’s Alpha and KOM of each latent variable are all above 0.813 and 0.721, respectively, which meet the above judgment criteria. This indicates that the reliability and validity of the questionnaire are reliable and reasonable, and the next step of model analysis can be carried out.

**Table 3:** Reliability and validity analysis of questionnaire.

Latent variable	Land-use	Pedestrian street	Public service	Green space	Public transportation	Individual conditions	Dietary habits	Exercise habits
α value	0.931	0.907	0.886	0.873	0.892	0.871	0.868	0.813
KOM value	0.856	0.853	0.745	0.721	0.734	0.756	0.729	0.772

### 2.4 Research framework

**Figure 4:**
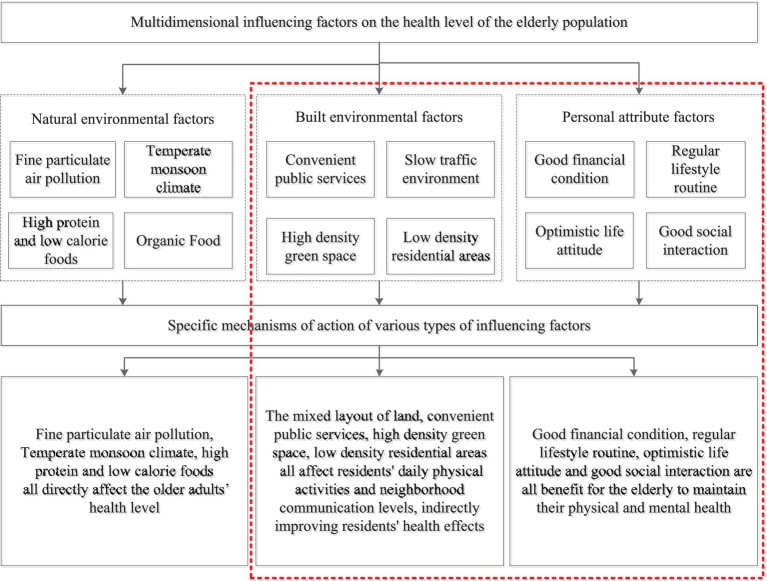
The classification of block-scale built environment elements.

Previous studies have shown that the main factors influencing resident health in the built environment include natural, built, and neighborhood environments (44). Residents’ health is not only influenced by factors such as individual socioeconomic background, dietary habits, fitness, and exercise habits but also constrained by environmental factors of higher-level block construction (48). The built environment mainly affects residents’ daily physical activities and convenience of accessing public resources, indirectly affecting their physical health (49). At the neighborhood level, this is mainly through cultivating good daily routines and dietary habits, promoting neighborhood communication among older adults, and indirectly improving their physical and mental health (Figure 4).

Due to the factors that affect residents’ health level, including multiple dimensions, such as material environment, activity behavior, and personal attribute differences, multi-level structural characteristics are present. We divided the explanatory variables into two levels: block and individual. We explore the relationship between the built environment and the older adults’ healthy behavior from three aspects: block-scale built environment, individual basic attributes, and daily living habits.

### 2.5 Models and methods

Traditional regression models, such as logistic regression, have been used for simple factor analysis. The single-level model underestimates the standard error of the regression coefficients of the variables and overestimates their significance (43). Hierarchical linear models (HLM) are statistical analysis techniques used to analyse data with nested structures that can effectively influence organizational or background effects (44, 45). The effects of explanatory variables at different spatial scales and management levels on the dependent variables are separated by establishing regression models for different levels of data. The advantage of this method is that it can distinguish the group effect from the individual effect by dividing the variation in the explanatory variable into intra-group and inter-group differences. This is helpful in revealing the relationship between the group and individual variables (46, 50). In addition, compared with traditional linear regression models, hierarchical linear models have more advantages in terms of parameter estimation methods and algorithms, model assumptions, and data requirements. Compared with the traditional linear regression model, the ordinary least squares (OLS) method is used to estimate the parameters. The hierarchical linear model uses the expectation maximization method (48, 49), Fisher scoring method (46), and iterative general least squares method to create a stable and accurate parameter estimation (51, 52). The hierarchical linear model does not require an independent normal distribution or homogeneous variance for random errors; therefore, it has wider applicability. As the iterative algorithm is used to estimate the parameters, the variance and covariance results can be effectively estimated when the data are unbalanced. When dealing with hierarchical data, the model first establishes a regression equation for the first layer of characteristic variables. The intercept and slope in the equation are considered dependent variables, and the characteristic variables in the second-level data are considered independent variables to be replaced by the model for quadratic regression. The basic formula is as follows (Equations 1-3):

First level:


(1) $$Y_{ij}=\beta_{0j}+\beta_{1j}\left(X_{ij}-\bar{X_{ij}}\right)+\gamma_{ij}$$

Second level:


(2) $$\beta_{0j}=\gamma_{00}+\gamma_{01}W_{j}+\mu_{0j}$$

(3) $$\beta_{1j}=\gamma_{10}+\gamma_{11}W_{j}+\mu_{1j}$$

**Figure 5:**
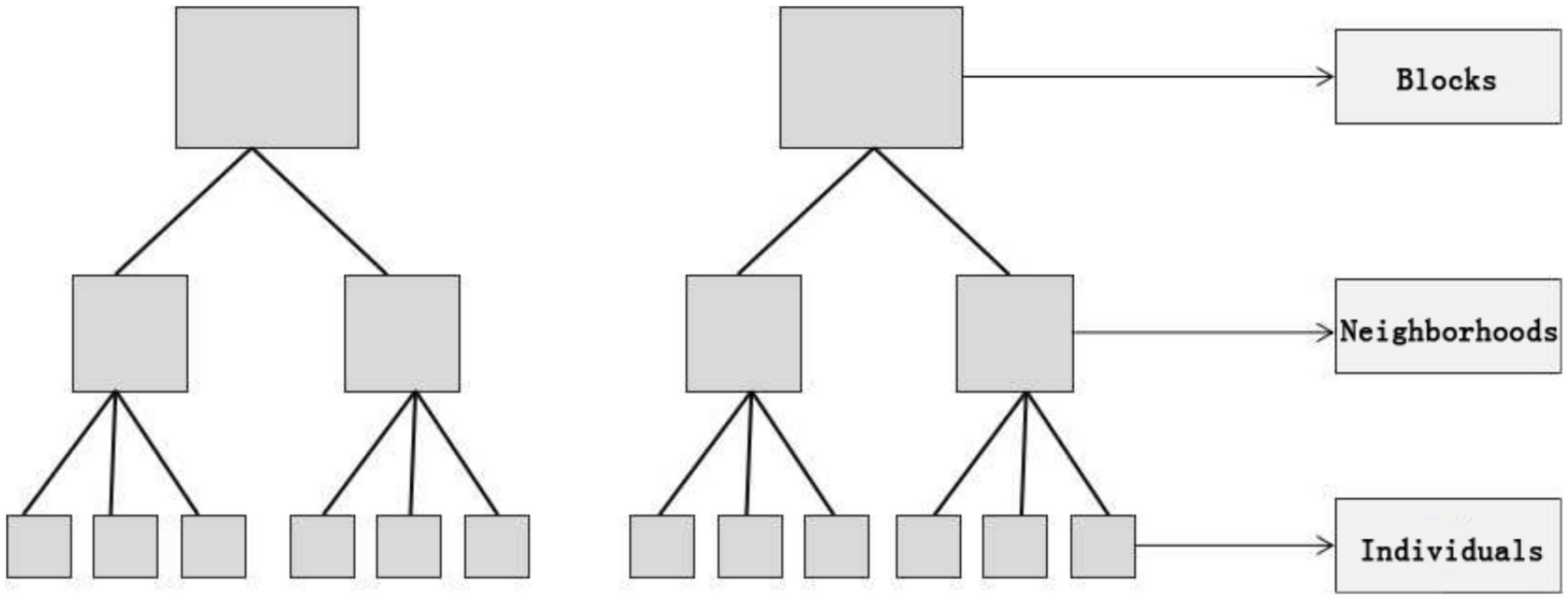
Schematic representation of the hierarchical structure of the dataset.

*W_j_* and *X_ij_* are characteristic variables for the first and second layers; *μ*_0*j*_, *γ_ij_*, and *μ*_1*j*_ are random effect variables. The feature variables of the first layer were treated as total or group averages. The interception β_0_ is the average value of *Y_ij_*, and the slope *β*_1*j*_ is the change amount that was produced by upgrading a unit of *X_ij_*. Characteristic variable *W* affects dependent variable *Y* by affecting the slope and intercept in the regression equation of the first-level characteristic variable at the second level. In the initial iteration stage, the multi-level linear model decomposes the dependent variable variation into two parts, intergroup and intragroup differences, and then identifies the relationship between individual and organizational variables (Figure 5).

We perform a fit test on the HLM and adjust the fit index based on the MI value until it meets the prediction criteria. The fitness index values of the model are shown in Table 4, and the measured values are all within the standard range, indicating that the model has good fitness and can effectively analyse the interaction effects between various latent variables (36).

**Table 4:** Model fitness index.

Adaptability index	X^2^/df	GFI	AGFI	RMESA	SRMR	AIC	BIC
Standard scope	1 ~ 3	>0.9	>0.9	<0.08	<0.08	1.19	1.32
Actual measured value	1.331	0.972	0.964	0.013	0.042	/	/
Adaptability judgment	Yes	Yes	Yes	Yes	Yes	/	/

Among them, the Goodness of Fit Index (GFI) ranges from 0 to 1, and the larger the value, the better the fit. GFI is used to measure the degree of agreement between sample data and theoretical models. The Adjusted Goodness of Fit Index (AGFI) is a modified GFI that reduces the impact of model parameters on model fit through penalty terms and can be used to evaluate model fit. Root Mean Square Error of Approximation (RMSEA) is the mean square error approximation degree, which ranges from 0 to 1. It is generally considered that a RMSEA below 0.05 indicates excellent model fitting, between 0.05 and 0.08 indicates good model fitting, between 0.08 and 1 indicates poor model fitting, and above 0.1 indicates extremely poor model fitting. Standardized Root Mean Square Residual (SRMR) is a standardized variance residual. The value ranges from 0 to 1, with smaller values indicating better model fit. It is generally believed that a SRMR below 0.08 indicates better model fit. Akaike Information Criteria (AIC) and Bayesian Information Criteria (BIC) are standards for measuring the goodness of fit of statistical models. The smaller the AIC value, the simpler the model and the better the fitting effect. AIC less than 2.1 standard deviations is acceptable, while AIC less than 1.2 indicates good model fit. AIC greater than 3 indicates poor model fit. We used cross validation techniques to verify the fit of the model, and the data results showed that the model fit was good and there was no overfitting phenomenon.

We use discriminant validity matrix analysis to demonstrate whether there is a correlation between latent variables and their corresponding observed variables. The testing standard of the model is whether the overall correlation (square root of AVE) between a latent variable and its corresponding multiple observed variables is greater than the correlation between the latent variable and other latent variables, that is, the absolute value of the Pearson correlation coefficient. If the value is greater than other values, it indicates good discriminant validity between the latent variables (37). The square root values and Pearson coefficients of AVE involved in this article are shown in Table 5, and they meet the criteria for discriminant validity testing.

**Table 5:** Discriminative validity.

Latent variable	Land-use	Pedestrian street	Public service	Green space	Public transportation	Individual conditions	Dietary habits	Exercise habits
Land-use	**0.806**							
Pedestrian street	0.033	**0.842**						
Public service	0.002	0.008	**0.835**					
Green space	0.326	0.117	0.017	**0.845**				
Public transportation	0.178	0.163	0.019	0.073	**0.866**			
Individual conditions	0.172	0.083	0.027	0.118	0.116	**0.858**		
Dietary habits	0.191	0.122	0.037	0.057	0.098	0.127	**0.839**	
Exercise habits	0.277	0.173	0.008	0.378	0.217	0.336	0.319	**0.822**

The bold values on the diagonal represent the root of AVE, whereas the values below the diagonal represent the Pearson correlation index between latent variables.

## 3 Results

### 3.1 Spatial heterogeneity in the built environment quality of different blocks

**Figure 6:**
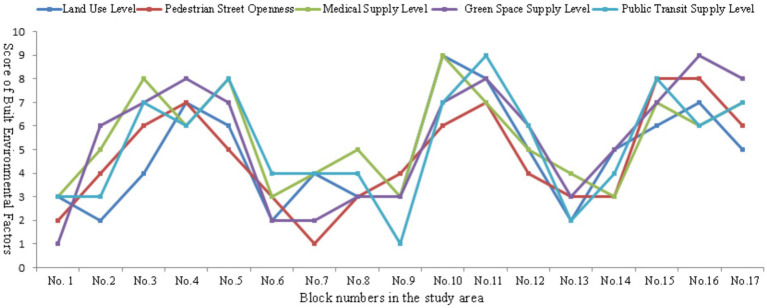
The evaluation of five built environmental factors on block scale.

This study used the data entropy method to calculate the impact weights of each built environment element and quantified and scored five types of built environment elements in 17 blocks (Figure 6). Overall, each block had a high level of construction in terms of land use diversity, public service resource levels, and green space resource supply, whereas the scores for road openness and public transportation resource construction quality were low. The statistical results showed that neighborhoods of older adults had relatively complete service facilities, whereas significant differences were observed in the quality of external travel and pedestrian environment construction within the neighborhoods, resulting in low road connectivity. From the perspective of spatial patterns, the scores of built environmental factors in each block showed a characteristic pattern of decreasing with each layer of the circle. The scores of the built environmental factors in blocks in the central urban area were generally higher, indicating that the comprehensive supporting facilities were relatively complete, whereas the quality of the built environmental configuration in the outer blocks was uneven. Two main reasons were identified for this spatial heterogeneity. The first was the guiding role of urban planning. Due to limited resources, to achieve the coordinated development of the entire city, the government set differentiated development goals and positioning for different streets within the city in urban planning decisions. Streets with different developmental positioning requirements receive different development resources, leading to different built environment outcomes. However, due to spatial differences in land prices, various commercial activities and public services in cities gathered in different locations, and streets located in different locations of the city exhibited heterogeneity in the quality of the built environment.

Higher diversity index of land use in blocks was associated with higher road connectivity and supply of public transportation facilities. This type of block was mostly located in the bustling commercial areas of the city and presented mixed land layout characteristics, such as catering, shopping, and accommodation. Moreover, neighborhoods with better public service resources had a higher supply of green space resources in their surrounding parks. The average score for the five elements of the built environment in neighborhoods with frequent neighborhood interactions and active walking activities was relatively high, whereas the opposite was relatively low. The results revealed a short board effect in the block-scale built environment. Blocks with good built environments had good scores for all indicators, whereas those with poor scores had poor scores for all indicators. In addition, the results reflected that the heterogeneity of urban block facility configuration and construction quality affected residents’ daily lives and behavioral habits, thereby affecting their health. This conclusion confirmed the rationality of using a multi-level model for the stratified analysis of influencing factors in this study. The heterogeneity between blocks reflected multiple aspects, such as land use, spatial form, facility layout, and population attribute characteristics. Two main reasons were identified for the heterogeneity. The first was the guiding role of urban planning. In urban planning decisions, the government established different development positions for different streets for the coordinated development of the entire city.

Furthermore, the establishment of a land market guided the generation of heterogeneity. Due to spatial differences in land prices, the behavioral activities of different types of residents gathered in different locations, resulting in heterogeneity in neighborhoods situated in different locations of the city. Compared to existing studies, some studies have found a significant positive correlation between mixed land use and walking among older adults through a combination of subjective and objective measurements (44). The presence of more than two types of land use in the neighborhood, including entertainment and commercial types, can encourage older adults to leave their homes and engage in daily walking activities or go to the park to exercise (45). Commercial service facilities within the neighborhood, such as bookstores, vegetable markets, and shopping venues, can effectively encourage older adults to use their leisure time to get out and walk, thereby meeting their daily needs and potentially increasing leisure physical activity (43). A study found that older adults living in high land-mix neighborhoods were more likely to increase walking time and frequency than those living in single land-use neighborhoods (44).

### 3.2 Significant differences in walking activity levels within different spatial levels

The model first introduced individual-level variables and then block-level variables. Due to the significant correlation between land use, street openness, public service supply, green space supply, and public transportation convenience among the block-level variables, five types of variables were introduced into the model separately to avoid the instability of model estimation results caused by collinearity. As the variance decomposition coefficient can measure the explanatory power of inter block differences on dependent variables, before introducing variables, a null model without introducing any explanatory variables was established to calculate the variance between individuals and block levels, resulting in a variance decomposition coefficient of 31.2%. The differences in residents’ health levels were mainly due to the heterogeneity at the individual level, and the heterogeneity at the block level impacted health differences. After introducing block-level variables into Models II, III, IV, V, and VI, a significant decrease in DIC was observed, indicating that the model had better explanatory power for residents’ health levels after introducing block level variables. Deviance information criteria (DIC) were used to measure the fitness, goodness of fit, and complexity of a model. The smaller the DIC value, the better the performance of the model.

The model analyzed differences in the walking activity levels of older adults in different spatial units within the city and the magnitude of the differences. Specifically, a multi-layer linear model was used to extract the interpretable variance proportion of pedestrian activity within the two spatial levels of blocks and residential areas. Table 6 presents the model *α* with two levels of individuals and blocks and *β* variance estimation results of two empty models, with three levels of individuals, residential communities, and blocks. Among them, the proportion of variance at the residential community level was small (6.8%), whereas the proportion of variance at the neighborhood level was relatively large (26.7%). Table 7 presents the differences found in model β in probability between the lowest and highest predicted values of the two high-level units and the average health level of residents in the residential areas with the worst and best health levels. At the street level, the differences indicated that the heterogeneity at the street level is higher than that at the residential community level. Considering the small variance at the residential community level, no significant differences were found in walking activity levels among older adults at the community level, verifying the rationality of the study’s selection of street and individual levels.

**Table 6:** ANOVA estimation results of hierarchical linear model for residents’ BMI.

Model	Individual variance ratio	Block variance ratio	Residential area variance ratio	DIC/pD
Model α (2 levels)	2.813 (68.8%)	1.276 (31.2%)	NA	4,235/178
Model β (3 levels)	2.537 (64.2%)	1.132 (28.6%)	0.284 (7.2%)	4,229/165

Deviance information criteria (DIC) were used to measure the fitness, goodness of fit, and complexity of a model. The smaller the DIC value, the better the performance of the model.

**Table 7:** Probability predictive value of high-level unit in Model β.

Model		Minimum (residential area)	Maximum (residential area)	Minimum (block)	Maximum (block)
Model β	Standard shape	23.7%	31.2%	14.8%	39.6%
	Slightly unbalanced	25.6%	20.9%	31.4%	17.2%
	Obviously unbalanced	35.2%	28.5%	40.5%	22.7%
	Highly unbalanced	10.4%	12.6%	8.4	9.6
	Extremely unbalanced	5.1%	6.8%	4.9	10.9

### 3.3 The synergistic effect of the block-scale built environment and walking activities on the health of older adults

**Figure 7:**
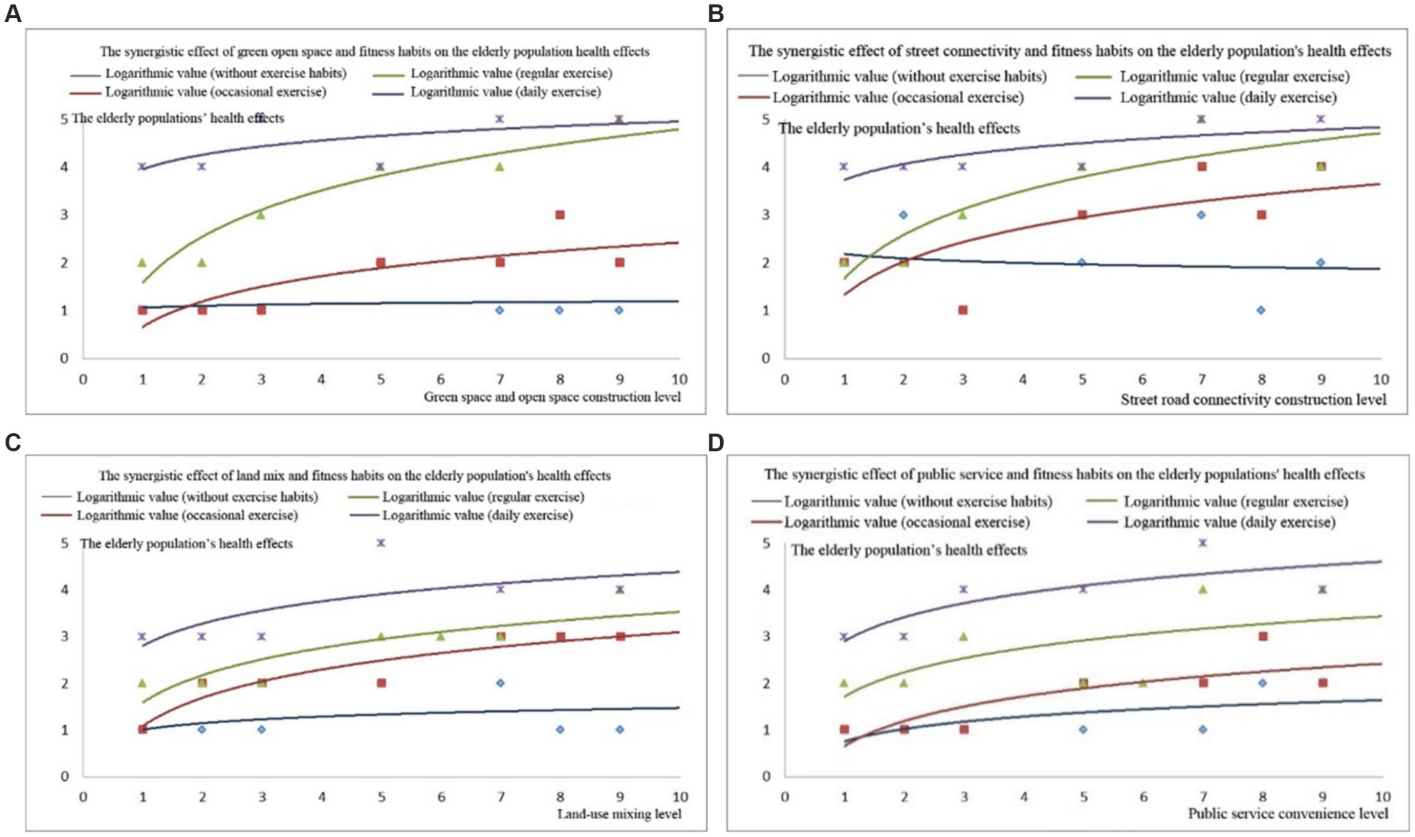
Health effects of green space accessibility: **(A)** road connectivity, **(B)** land use mix, **(C)** and public service convenience, **(D)** effects on older adults in relation to their walking habits.

Figure 7 shows the health effects on older adults under different walking frequencies, which were significantly positively correlated with indicators, such as the accessibility level of street green spaces and the length of pedestrian streets. The results showed that the sample population, including the green space accessibility and pedestrian activity scores, had an overall threshold in a discrete distribution state. Therefore, we used logarithmic regression trend lines to fit the trend of the data changes (50). As shown in Figure 7A, the health of older adults with different walking activities was positively correlated with the accessibility indicators of the surrounding green spaces. Among them, older adults who never exercised maintained a relatively low level of health and did not show significant improvement or decrease in accessibility to residential green spaces. The health of older adults who occasionally exercised increased with accessibility to green spaces. The health of people who exercised regularly exhibited a significant improvement with improvements in green space accessibility. In addition, the results showed that in the initial stage of increasing accessibility to green spaces, the health of older adults increased rapidly, indicating that within the functional radiation range of green parks, residents use green space facilities more frequently, which can produce good health. The health of residents who exercised daily was maintained at a high level, and the accessibility indicators of green spaces had a relatively impact on their health (Figure 7).

The mileage of pedestrian streets reflected the environment (Figure 7B). The health of older adults who never exercised or exercised regularly did not change significantly with an increase in walking distance on the street. In the occasional and frequent exercise groups, walking distance in the neighborhood had a significant impact on health. The results indicated that this group of people effectively used pedestrian streets in the neighborhood, improving their health. This conclusion is consistent with research in regions such as the United States and Australia, where a higher neighborhood walking index is more conducive to increased adult walking activity. In addition, empirical studies in Europe have found that an improvement in the neighborhood walking index can effectively improve the health of residents (48, 49, 51).

The result shows significant positive correlation between neighborhood walkability and the enhancement of walking frequency and activity. For every 10% increase in pedestrian road connectivity and land use, the walking activity volume increased by 22.4 and 12%, and the BMI increased by 9.1 and 7.3% toward the standard range, respectively. For every 10% increase in distance between residence and plaza and park, the walking activity volume decreased by 5.4 and 3.2%, BMI decreased by 9.9 and 6.3%, respectively. For every 10.4% increase in land mixed-use rate and *per capita* green area, BMI increased by 19.4%. A study on the relationship between pedestrian suitability, street environment, and physical health found that for every 10% increase in the distance between residential areas and rivers, parks, or squares, the overweight and obesity rates of residents increased by 14.4, 18.7, and 19.6%, respectively (49). The results indicated that for every 10% increase in physical activity as an intermediate variable, BMI increased by 3%, indicating a correlation between the residential environment and residents’ physical health (51).

## 4 Discussion

### 4.1 The block-scale built environment had a significant impact on the walking activity of older adults

According to the results of models II, III, IV, V, and VI (Table 8), the land use, road walkability, public service supply, green space supply, and public transportation convenience at the block level impacted the walking behavior of older adults in the block. First, the impact of land-use diversity indicators was positive, indicating that more types of resource services and higher degree of land mix within the block were associated with better health of residents. Previous studies have argued that commercial and public service facilities, as well as land mix, are important components of the walkable environment in neighborhoods and that built environment factors promote walking activities (48). Previous studies have shown that mixed land use in community neighborhoods can shorten residents’ travel distances and create conditions for active travel between destinations. These factors can increase the walking activity of middle-aged and older adults (49) and have a positive impact on children’s moderate-to high-intensity walking activity (50), thereby improving health. Furthermore, the impact of the residential density indicator was negative, indicating that higher residential density as associated with lower willingness to walk. An overly dense residential environment can reduce residents’ occupancy of public facilities and green spaces in the neighborhood, *per capita* facility occupancy and usage levels, and residents’ willingness to walk. This is not conducive to the formation of residents’ daily health exercise habits, thereby damaging health. Moreover, the mileage of pedestrian roads, road density, and road connectivity had significant positive impacts. Research has shown that open streets are more conducive to leveraging the functions of public spaces and facilitating residents’ physical activities and social interactions (42). In addition, open streets generally belong to the land-use model of mixed commercial and residential development, which is conducive to residents performing transportation and leisure walking activities. Some studies have found that better street connectivity was associated with shorter distance between departure and destination, which increased residents’ willingness to walk and exercise (43). Unlike existing research, this study found that the transportation system had a significant impact on the walking activity of older adults. The main influencing factors were distance to the transportation destination, type of street network, and road connectivity (44). Among these, the connectivity of the street network had a significant positive impact on walking and cycling, and the connectivity of the surrounding streets in residential areas was positively correlated with pedestrian traffic activity (48).

**Table 8:** The results for hierarchical model of block environment variables.

	Model I	Model II	Model III	Model IV	Model V	Model VI
**Constants**	54.74*** (1.433)	57.69*** (1.997)	63.22*** (2.512)	39.94*** (5.383)	40.14*** (5.552)	61.28*** (1.712)
**Built environment variables**						
Residential density		−0.394* (0.323)				
Land-use mix		5.941** (1.512)				
Pedestrian street mileage			3.354*** (1.012)			
Pedestrian street density			1.954** (0.319)			
Pedestrian street connectivity			4.578*** (1.443)			
Number of public service facilities				0.431 (0.232)		
Accessibility of public service facilities				2.194** (0.997)		
Number of green parks					0.134 (0.182)	
Accessibility of green park					3.168** (1.147)	
Number of public transport facilities						1.064* (0.712)
Accessibility of public transport facilities						1.492** (0.842)
**Individual variables**						
Age	−0.254*** (0.209)	0.243 (0.198)	−0.257 (0.211)	−0.292 (0.235)	−0.231 (0.221)	−0.244 (0.215)
Age squared^①^	−0.382** (0.176)	−0.352 (0.169)	−0.391* (0.174)	−0.388** (0.172)	−0.386 (0.172)	−0.387** (0.181)
**Gender (reference group: female)**						
Male	0.397 (0.647)	0.382 (0.639)	0.365 (0.627)	0.373 (0.648)	0.373 (0.626)	0.392 (0.655)
**Education level (reference group: university)**						
Junior high school and below	−0.189 (1.043)	−0.096 (1.066)	−0.182 (1.326)	−0.161 (1.226)	−0.135 (1.071)	−0.115 (1.037)
Senior high school	0.448* (0.790)	0.434* (0.805)	0.452* (0.919)	0.428* (0.785)	0.473* (0.794)	0.335** (0.794)
Postgraduate and above	−0.089 (1.319)	−0.108 (1.861)	−0.107 (1.324)	−0.083 (0.923)	−0.092 (0.992)	−0.117 (1.253)
**Marital status (reference group: unmarried)**						
Married	0.497 (0.547)	0.482 (0.539)	0.475 (0.527)	0.473 (0.548)	0.473 (0.548)	0.490 (0.555)
**Monthly family income (reference group: <4,000 yuan)**						
4,000–8,000	0.899 (1.014)	0.886 (1.002)	0.766 (0.930)	0.850 (0.992)	0.739 (1.006)	0.842 (0.962)
8,000–12,000	3.096** (1.394)	2.873** (1.375)	2.821** (1.335)	2.927** (1.386)	2.862** (1.336)	2.832 (2.362)
Above 12,000	2.848** (1.527)	2.624** (1.547)	2.425** (1.43)	2.547** (1.443)	2.469** (1.422)	2.534* (2.206)
**Daily life habits**						
**Dietary habits (reference group: balanced nutrition)**						
Unhealthy diet (excess on salt, oil, sugar)	−3.162** (1.229)	−3.011*** (1.238)	−2.934** (1.206)	−2.921*** (1.335)	−3.096** (1.394)	−2.872** (1.375)
**Physical activity (reference group: never exercise)**						
Exercise every day	1.721** (1.017)	1.808** (1.081)	1.624** (0.981)	1.719** (1.031)	1.677** (0.997)	1.737** (0.925)
Exercise regularly	0.899* (1.023)	0.871* (0.906)	0.824** (1.015)	0.931** (1.147)	0.927** (1.205)	0.882* (1.062)
Exercise occasional	−0.189 (1.043)	−0.027 (1.066)	−0.082 (1.066)	−0.061 (1.086)	−0.045 (1.071)	−0.335 (0.794)
*pD*	57925.652	57931.324	57925.715	57927.446	57929.871	57925.685
*DIC*	49.265	41.732	41.425	42.568	40.652	42.329

① Age square of centralized processing, with a value of (Actual age – Mean age)^2^. *, **, and ** significance level of 90, 95, and 99%, respectively. Logarithm of numerical variables at block level.

In addition, no significant correlation was observed between the number of public service and park facilities, whereas the accessibility indicators of public service facilities and park green spaces showed a significant positive correlation. Previous studies have shown that public open spaces can provide a low-cost activity environment and are important destinations and venues for daily leisure walking among people of different ages and income groups. Quantity, accessibility, internal environmental design, quality, and other factors determine the frequency of resident use, thereby affecting their level of walking activity (48). Australian scholars have found that adults who visit parks more than once a week have a 26% increased chance of participating in leisure walking and a 11% increased chance of participating in moderate-to high-intensity walking activities (53). Most existing research has focused on examining the relationship and mechanism between the quantity, quality, and accessibility of park green spaces and residents’ frequency of use and level of walking activity (44, 49). Some scholars found that factors such as the quantity, quality, and convenience of park facilities were positively correlated with overall walking activity, thereby significantly improving residents’ health (50). The number of public transportation facilities and accessibility indicators showed a significant positive correlation, indicating that the more public transportation facilities were arranged within the block; closer distance to residential areas was associated with higher health index of the block. A possible reason for this is that the high-density bus stop layout facilitates residents’ daily travel, cultivates slow travel habits, and alleviates issues such as the lack of physical activity caused by motor vehicle travel. Unlike existing studies, this study identified a significant positive correlation between the level of street greening and public service facilities and residents’ walking activities. The presence of parks and entertainment venues had a positive effect on physical activity among older adults (48). Scholars have identified a significant positive correlation between the density of park green spaces and the probability of leisure walking activities lasting more than 1 h per week (49). Some scholars have argued that a higher density of parks can increase physical activity and the availability and accessibility of parks and enable older adults to use parks for walking (51).

The results indicate that the built environment of the community has a positive impact on the health of older adults. Regarding the built environment, the distance of pedestrian roads, connectivity, and land use mix have the highest significance and are the main factors influencing older adults’ health. This result is consistent with previous studies, which found that good facility accessibility has a promoting effect on health. Communities with high facility accessibility have relatively complete public transportation facilities, and various types of public facilities are evenly distributed with a high degree of mixing (31). The complete built environment is conducive to older adults’ engaging in local activities, including physical activities such as walking and cycling, thereby promoting improved health conditions (29). Studies have shown that older adults who are exposed to low-density or commercial decline environments for a long time have a higher risk of poor health compared with those who are in ordinary environments (20). Additionally, older adults who have difficulty accessing healthcare services face greater risks of health damage. When indoor environments are easily accessible, facility services are available and within reach, and safety is high, older adults’ mobility will be higher (23).

### 4.2 Socioeconomic attribute factors had a significant impact on walking activities of older adults

Among individual-level variables, monthly household income had a significant impact on walking activity. Older adults with a monthly household income of 8,000–12,000 yuan had a significantly higher walking activity level than the group with a monthly household income of less than 4,000 yuan. However, participants with incomes above 12,000 yuan did not show a significant positive correlation. Some low-income groups have limited economic capacity and insufficient attention to fitness behavior, which limits their access to public services. Therefore, the walking activity volume of low-income groups was relatively low. Furthermore, low-income groups face greater economic pressure and place greater emphasis on their daily survival needs. This group has a relatively low pursuit of quality of life, and their participation in walking and fitness activities is relatively low. Places such as parks and green spaces have weaker impacts on this group. We analyzed the possible nonlinear effects of age and introduced age and squared age values of centralized processing into the model. The results indicated that age and age squared simultaneously affect residents’ walking behavior. Older adults generally have low health awareness and lack fitness habits (52).

In addition, married individuals engaged in significantly more walking activities than unmarried individuals. Due to the stable family structure of married people, cooking at home was more beneficial for residents to obtain a healthy diet, and the care of partners enhanced their willingness to walk. Compared to a balanced diet, unhealthy dietary habits had a significantly negative impact on residents’ health. The lack of meat in the diet leads to insufficient protein intake, whereas excessive meat intake can lead to chronic diseases, such as hyperlipidaemia. Unhealthy eating habits, such as salt, oil, and sugar intake, can disrupt the normal metabolic rate of the body and increase the risk of obesity and chronic diseases. Compared with residents who never exercise, good walking and fitness habits helped older adults maintain a healthy state. The health status of people who exercised every day was generally better and gradually improved with the increase in fitness frequency, indicating that strengthening residents’ daily exercise activities can enhance their health.

The direct impact of individual attributes on the health of the older adults is 0.32, with income and age being the main factors affecting their health. Some studies suggest that economic income indirectly has a positive impact on the self-evaluation, as well as physical and mental health of older adults through physical exercise, with indirect effects of 13.5, 14.1, and 11.6%, respectively. Consistent with the results of this study, economic income has a positive impact on the health of older adults (39). Economic income also has a positive impact on physical activity among older adults, with high-income seniors actively participating more in physical activities (35). The possible reason is that physical activity mainly relies on disposable time; compared with high-income older adults, low-income older adults have relatively less disposable time, resulting in low physical activity (30). In addition, high-income people invest more in fitness, leisure, and entertainment, which increase their physical activity and promote their health status (31). The impact of socio-economic attributes on residents’ walking activity can be understood from the following two aspects: first, obtaining more flexible resources means having better material conditions and living environment and having more control over free time. Adequate free time is conducive to residents engaging in fitness activities such as walking (38). Second, elastic resources can be used in different manners under different circumstances. When the climate environment in a city deteriorates, high-altitude groups always have various strategies and ways to flexibly utilize various resources to avoid risks and achieve health protection (39). Some Chinese scholars’ research conclusions and viewpoints in this field are basically consistent, showing that older adults with higher education levels, higher incomes, and working within the system have better walking activity and health levels (40).

### 4.3 The built environment and individual attributes of the block had a synergistic impact on the walking activities and health of older adults

**Figure 8:**
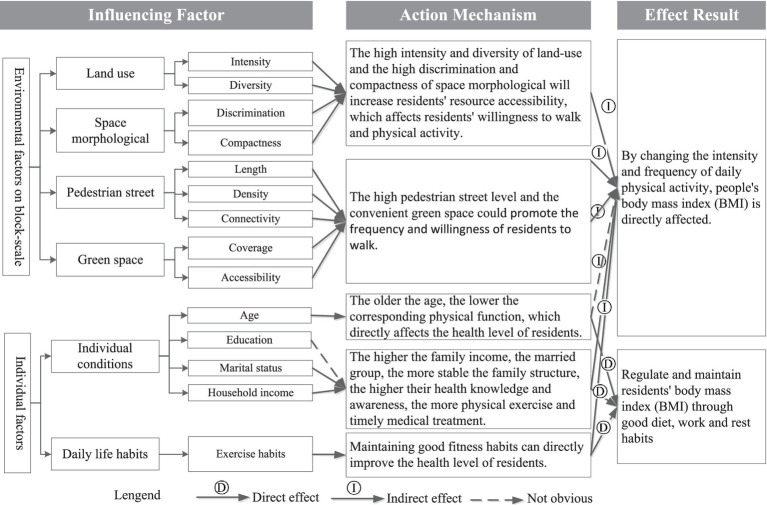
Influencing factors and mechanism of residents’ health level at block scale.

An interactive relationship was observed between the built environment and residents’ subjective cognition. Indicators such as the quantity, quality, and accessibility of various facilities in the built environment provided a material space environment for residents to develop their willingness to walk and exercise (Figure 8). Moreover, the socioeconomic background of residents significantly affected the development of their daily fitness habits, indirectly changing their frequency and willingness to use facilities. Specifically, land development intensity, road openness, public service supply, green space supply, and availability of public transportation facilities directly affected the frequency, accessibility, occupancy rate, and other indicators of older adults’ use of facilities, thereby affecting their willingness and intensity of daily walking activities and ultimately affecting their health. In addition, factors such as socioeconomic background and daily living habits indirectly affected the awareness and physical fitness of older adults, thereby affecting the frequency and health effects of walking for fitness. Higher density of residential buildings was associated with significantly lower resource occupancy rate of unit facilities, thereby significantly reducing individual access to resources and facilities and walking willingness. Furthermore, more diverse types of facilities were associated with higher openness of roads and higher density of green spaces, which effectively reduced the spatiotemporal distance of activities and promoted the active walking activities of older adults. Higher convenience of public transportation and higher supply level of public service facilities were conducive for older adults to engage in daily walking activities, such as seeking public service treatment, which positively affected their willingness to walk. Unlike existing research, the urban transportation system and land-use layout were unified and interacted with each other. Public service facilities within a reasonable distance of residential areas effectively increase the amount of physical activity related to transportation among residents (30). However, some studies found a significant negative correlation between road connectivity indicators and residents’ walking activity index (47). Although higher road connectivity can improve travel convenience, older adults may experience higher traffic safety risks, leading to a resistance to walking activities (38). Therefore, non-motorized transportation modes should be a key focus in block-scale transportation planning of modern urban transportation systems. Walking can be a good way to improve public health and encourage people to develop good daily living habits (39).

In terms of socioeconomic factors, age was positively correlated with a decrease in physical function and in willingness and frequency of walking. Higher family income and more stable marital status were associated with better living habits, which were conducive to walking activities, thereby improving health. This was in line with the findings of previous studies (38–40, 47). Rapid urbanization significantly changed the daily living environment and living habits of residents. Excessive dependence on motor transport, substantial reduction in personal physical activity, prevalence of sedentary lifestyle, and other causes of insufficient walking activities have led to obesity, cardiovascular diseases, diabetes, and other chronic diseases. After nearly a century of practice, developed countries have improved their urban environments by actively planning land use and transportation, providing open public spaces, building design opportunities for daily exercise, and increasing residents’ daily walking activities, thereby reducing health risks.

The results indicate that the built environment and individual attributes play a positive guiding role in the health level of older adults, to some extent promoting the investment in physical activity and transforming it into a return on health. Some studies suggest that good accessibility of facilities is beneficial for promoting physical activity among older adults and helping to alleviate the decrease in walking volume that occurs with age (40, 42). There is a close relationship between street connectivity, mixed land use, number of transportation stations, and physical activity among older adults. The better the connectivity, the more shops there are, and the more complete the service facilities are, the more important it is to promote physical activity (47). In addition, a compact land development model can enhance the vitality of street life and the support capacity of neighborhood commerce, generating greater attraction for older adults to engage in outdoor activities (44). Therefore, it is possible to improve facility accessibility and diversity design, actively plan or transform community fitness environments, promote an increase in physical activity, and thus have a positive impact on health.

Overall, the model of the impact of environmental factors at the block scale on the walking activities and health effects of the older adult population includes two aspects: maintaining their physical function and external activities. The first aspect is establishing and maintaining the internal physical functions of the older adult population, which helps individuals to mobilize all their physical and mental resources at any time, for example, reducing health risks, encouraging healthy behaviors, reducing potential barriers, providing functional services, and developing related abilities. The second aspect is providing environmental support, such as optimizing the daily living environment of the older adult population and reducing the negative externalities of the urban environment, for example, providing spatial spaces, social interactions, social policies, and other support systems. When the older adult population has a high degree of fit with the neighborhood environment, they can better maintain their physical functions and exert more social value. Studies have shown that the functional performance of the older adult population depends not only on their own physiological health level, but also on external constraints from environmental factors (31). When environmental support matches individual needs, the environment plays a positive supportive role in physical activity, and the functional performance of the environment is relatively high. However, when there is a mismatch between supply and demand, residents’ behavioral activities are suppressed and reduced to varying degrees (45).

**Figure 9:**
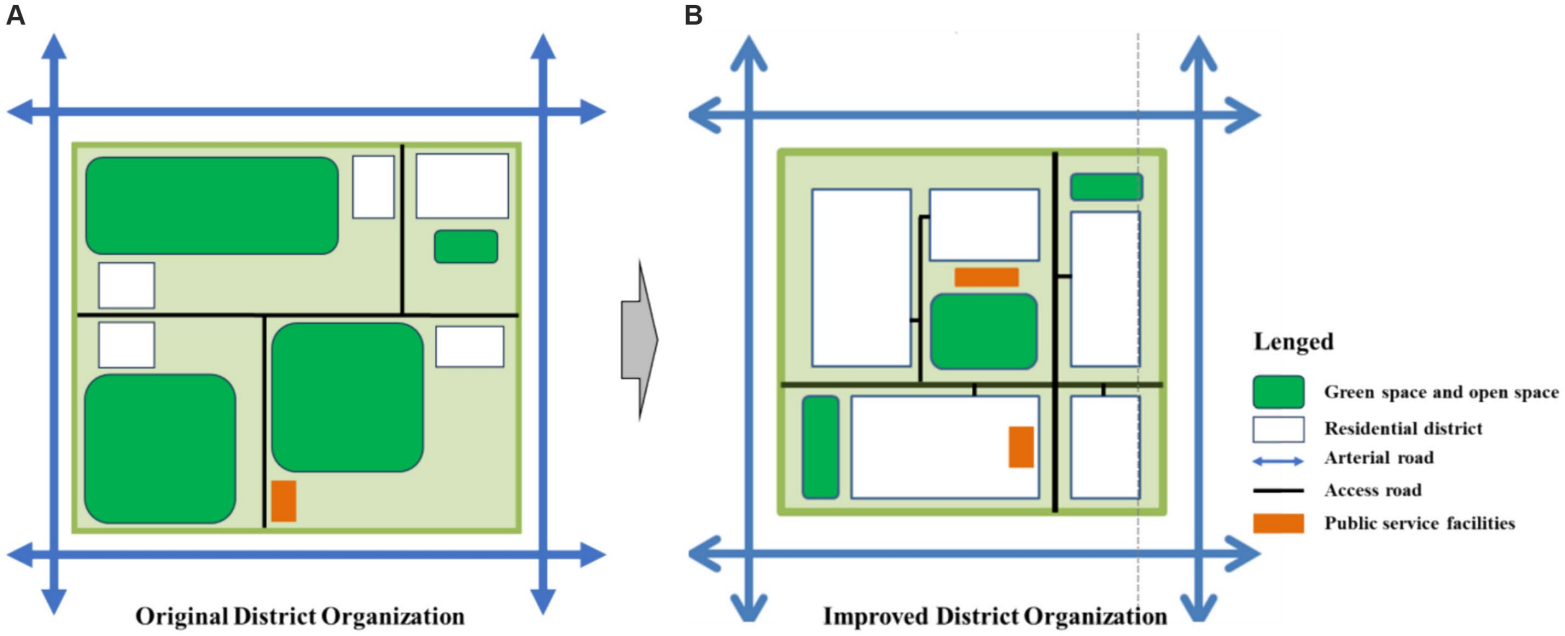
Enhancing residents’ willingness to engage in fitness activities by increasing the density of slow-moving road networks in neighborhoods. **(A)** Original District Organization; **(B)** Improved District Organization.

Our findings suggest that government management departments should improve the level and density of street networks in the renovation planning of old neighborhoods and promote residents’ daily fitness interaction. Urban roads are usually divided into four categories: expressways, main roads, secondary roads, and branch roads. High-level roads are mainly used for transportation functions in urban transportation to ensure high traffic capacity within the block, whereas low-level roads are mainly used for collection and distribution capacities, which need to be coordinated and balanced with other road functions (Figure 9). To ensure the normal and efficient operation of the overall road network, various levels of roads should cooperate to form a reasonable grading structure. The government is actively conducting road network encryption work, including increasing the number of low-grade urban roads and optimizing the grading structure of urban road networks (54). In addition, governments should reduce traffic obstruction caused by large enclosed blocks and improve and enhance urban traffic flow capacity by increasing the traffic permeability of the urban blocks (16, 47). Furthermore, form a continuous street network, governments should increase the coverage of public transportation stations through appropriate road network density and promote the use of green transportation methods, such as walking and cycling (42, 43). Moreover, governments should rebuild a humanized street space, shape a changing street interface, and improve the urban road landscape (39, 44).

### 4.4 The aging friendly renovation strategies for block pedestrian construction environments

Older adults mainly rely on walking for their daily outdoor activities. As they age, their physical functions, especially their visual, auditory, and motor abilities, significantly decline. Therefore, it is necessary to build a safe and convenient walking system for them. This article proposes that, based on the principle of integrity, the built environment of the block should be updated and transformed at multiple levels and in all aspects, including the following three aspects.

First, unblock the car dealership network. During the process of street design and renovation, classify and control motor vehicle traffic. Since the main external transportation roads are controlled by traffic lights, adopt restrictive measures for motor vehicles on other roads and clarify the areas and times for motor vehicle passage. Prohibit motor vehicles from entering during key time periods or provide alternative routes to enhance walking safety for older adults. By changing the road surface material and adding speed bumps and other measures, the speed of motor vehicles passing through the community can be reduced. The roads within the block should adopt a traffic organization method where pedestrians and vehicles are separated, and the corresponding road sections should be designed according to the diversion method.

Second, improve the walking system; promote the vitality guidance and flow of each space based on the connectivity and closeness of path connections between different venues within the block; increase the density of the road network appropriately based on the current pedestrian space; enhance the permeability with adjacent spaces, and thereby improve the continuity of walking for older adults. Use flat road surfaces, reduce height differences at road joints, adopt flat and uniform ground paving materials, and ensure the continuity and sufficient width of pedestrian roads as much as possible. Establish a continuous accessible pedestrian road system to ensure that older adults have barrier free access between residential entrances, pedestrian walkways, and activity areas. In addition, there should be clear and recognizable directional signs in the neighborhood to help older adults identify their destination.

Third, build a green system. In the renovation design, by revitalizing abandoned green spaces and converting vacant hard spaces into green spaces, community green resources are integrated to increase green coverage area. Based on older adults’ daily walking habits and the impact of urban built environment on walking activities, attention should be paid to subjective and objective accessibility, safety, and quality of use of public open spaces and service facilities such as green spaces, parks, schools, and bus stops. For example, moderately increase the accessibility of public open spaces and the connectivity between neighborhood parks to ensure that older adults’ can easily reach them by foot. Meanwhile, facilities such as interval rest areas and increased lighting supply should be set up on the park trails to facilitate older adults’ walking activities.

## 5 Conclusion

### 5.1 Key findings

The relationship between the built environment and residents’ health has become a hot topic of interdisciplinary attention in urban planning, design, and community governance. This study combined multi-source data and diversified survey methods, such as questionnaire surveys of older adults and surveys of built environmental facilities in blocks, and applied a multi-layer linear model to analyse the impact of different types of built environmental factors on walking activity and the health of older adults at the block scale.

The results revealed that indicators such as land mix, pedestrian street mileage, pedestrian street density, pedestrian street connectivity, distribution density of public transportation facilities, and public service accessibility within blocks were significantly positively correlated with walking activity volume in older adults. However, the residential density index in the neighborhood was significantly and negatively correlated with walking activity in older adults. Overly dense residential areas reduced the frequency and occupancy of fitness facilities and green spaces within blocks by older adults.

Furthermore, socioeconomic indicators such as family income, marital status, nutritional balance, and fitness habits were significantly and positively correlated with the amount of walking activity in older adults. However, age and the square value of age were negatively correlated with the amount of walking activity in older adults, making it difficult for older adults to effectively engage in walking and other fitness activities, and the fitness effect was relatively poor.

The built environment and socioeconomic attributes of the block had a superimposed effect on walking activity in older adults. Better quality of the built environment was associated with better family’s economic situation and higher willingness of older adults to engage in walking activities, resulting in better health. Family structure and income level were major constraints on older adults’ willingness to engage in walking. The various indicators of the built environment in blocks had nan indirect impact on the walking activity and health of older adults.

## 6 Implications

This study explored the impact of multi-level factors, such as built environment and lifestyle habits, on residents’ health at the block scale and empirically compared research in China and abroad, thereby enriching the literature in China. Residents’ health was influenced by multiple factors, such as the living environment, behavioral habits, and nature. The living environment of older adults and the surrounding neighborhood environment directly affected the daily life of older adults. The use of living spaces and daily habits by residents were the key factors that affected their health. Therefore, understanding the daily health behaviors of older adults requires not only objective measurement of environmental factors but also exploration of the perception and physical action in the environment (51). In addition, from the perspective of public health policy formulation, optimizing and organizing the built environment elements of neighborhoods is urgently required to enhance neighborhood social interactions and promote residents’ walking activities.

## 7 Limitations and future research directions

Governments should provide guidelines for the healthy living and daily habits of residents and construct the community environment to enhance the efficiency and intensity of the use of fitness facilities and pedestrian streets. Moreover, urban planning and public policy formulation should focus on the balanced creation of residents’ public activity space, allocate social resources reasonably based on the actual needs of residents’ activities, improve land use structure, compensate for the lack of public activity space in a capital-driven environment, and provide environments to improve individuals’ physical and mental health (31). Our empirical micro-level findings are in line with the characteristics of urban space and the background of socioeconomic transformation in China, which compensates for the limitations of applying theoretical research results from developed Western countries. This study provides a reference for creating healthy public spaces and public health policy at the district level in response to a rapidly aging society.

Studies have shown that air pollution and noise levels can affect the walking willingness and ability of older adults (52). People who inhale high doses of PM2.5 in the short term may experience difficulty breathing, coughing, runny nose, glare, sore throat, and palpitations (55). Older adults with potential health hazards face higher risks, such as asthma, chronic obstructive pulmonary disease, or heart disease. Because PM2.5 directly damages the respiratory tract, causing a large amount of inflammation and exacerbating their condition, older adults’ willingness to walk in polluted air environments is weakened (53). The traffic flow on main roads is high and the speed is fast, resulting in significant decibel noise pollution. Older adults’ willingness to walk in noise polluted environments is significantly reduced (56).

Body Mass Index (BMI) is a commonly used indicator to measure the degree of body fat, thinness, and health. It can be calculated by measuring weight and height, and the data acquisition method is simple and convenient (57). BMI can represent the total body fat and is closely related to the distribution of body fat and body fat percentage. Research indicates that BMI is closely related to a variety of chronic disease risks, such as hypertension, coronary heart disease, type II diabetes, and some cancers. An increase in BMI is closely related to an increased risk of various chronic diseases (58). For example, a meta-analysis showed that for every 5 kg/m^2^ increase in BMI, the risk of all-cause mortality increased by 20% (59). We will attempt to incorporate other health indicators such as functional activity ability, mental health, and cardiovascular health in future studies, which can provide a more comprehensive assessment (60, 61).

## Data Availability

The original contributions presented in the study are included in the article/supplementary material, further inquiries can be directed to the corresponding author.
